# Production of Autoantibodies in Chronic Hepatitis B Virus Infection Is Associated with the Augmented Function of Blood CXCR5^+^CD4^+^ T Cells

**DOI:** 10.1371/journal.pone.0162241

**Published:** 2016-09-09

**Authors:** Yu Lei, Tingting Hu, Xiaofei Song, Hong Nie, Min Chen, Weixian Chen, Zhi Zhou, Dazhi Zhang, Huaidong Hu, Peng Hu, Hong Ren

**Affiliations:** 1 Department of Infectious Diseases, Institute for Viral Hepatitis, Key Laboratory of Molecular Biology for Infectious Diseases, Ministry of Education, the Second Affiliated Hospital, Chongqing Medical University, No.74 Lin Jiang Rd., Yu Zhong District, 400010, Chongqing, People’s Republic of China; 2 Department of Laboratory Medicine, the Second Affiliated Hospital, Chongqing Medical University, No.74 Lin Jiang Rd., Yu Zhong District, 400010, Chongqing, People’s Republic of China; Harvard Medical School, UNITED STATES

## Abstract

T follicular helper cells (Tfh) provide help to B cells to support their activation, expansion and differentiation. However, the role of Tfh cells in chronic HBV infection is poorly defined. The aim of this research was to examine the function of Tfh cells and whether they are involved in HBV related disease. Blood CXCR5^+^CD4^+^T cells and B cells in 85 patients with chronic HBV infection (HBV patients) and health controls (HC) were examined by flow cytometry. The molecule expression in blood CXCR5^+^CD4^+^ T cells was detected by real-time PCR. Blood CXCR5^+^CD4^+^ T cells and B cells were co-cultured and the production of Ig and cytokines was detected by ELISA. Autoantibodies were detected by indirect immunofluorescence and immunospot assay. We found that blood CXCR5^+^CD4^+^ T cells in patients with chronic HBV infection (HBV patients) expressed higher level of activation related molecules and cytokines than that from health controls (HC).In HBV patients, the frequency of blood CXCR5^+^CD4^+^ T cells was significantly correlated with serum ALT and AST. We also found that blood CXCR5^+^CD4^+^ T cells from HBV patients could induce B cells to secret higher level of immunoglobulin than that from HC. Several autoantibodies, including ANA, ss-A, ss-B, Scl-70, Jo-1, ect, were indeed positive in 65% HBV patients. Among HBV patients, expression of function related molecules was significantly higher in blood CXCR5^+^CD4^+^ T cells from patients with autoantibodies than that without autoantibodies. Our research indicated that blood CXCR5^+^CD4^+^ T cells from HBV patients were over activated and show augmented capacity to help B cells for antibody secreting, which might correlated with liver inflammation and the production of autoantibodies in extrahepatic manifestations.

## Introduction

Hepatitis B virus (HBV) is a noncytopathic, hepatotrotic member of the hepadnavirus family that causes acute and chronic hepatitis, cirrhosis and hepatocellular carcinoma (HCC)[1, 2, 3]. In addition to liver diseases, acute, especially, chronic HBV infection is associated with a variety of extrahepatic manifestation that affect a variety of organs or tissues, including kidney, blood vessels, skin, and joints[3, 4, 5].One of the pathogenetic roles in the development of these extrahepatic manifestations is the production of autoantibodies (Ab), like anti-smooth muscle Ab, antinuclear Ab, anti-nucleosome Ab, anti–liver-kidney microsomal Ab, which leads to the lesion of responding organs and tissues[4–7].However, the pathophysiology and the full spectrum of immunological factors that involved in the HBV infection associated manifestation are not completely defined.

Many researches have suggested that a series of immune cells, including CD8^+^ T cells, CD4^+^ T cells, NK cells, B cells and γδ T cells are involved in the pathogenesis of HBV infection[8–12]. Recently, a distinct proportion of CD4^+^ help T cells present in germinal centers (GCs) was defined as T follicular helper (Tfh) cells[13, 14]. Tfh cells were characterized as high expression of chemokine receptor CXCR5 [15, 16], specific transcription factors Bcl-6 [17, 18],and producing cytokines, especially IL-21 and IL-4 [19, 20]. In GCs, Tfh cells provide signals including co-stimulatory moleculesCD40L,inducible co-stimulator (ICOS) [21], programmed cell death 1 (PD-1) [22, 23] as well as IL-21, IL-4 to B cells for their survival, differentiation and proliferation[19, 20].At the same time, B cells present antigen and provide co-stimulatory signals which maintain the phenotype of Tfh cells. In circulation, blood CXCR5^+^CD4^+^ T cells have been verified to be counterparts of Tfh cells from GCs with capacity to support antibody secreting by B cells [24, 25].

Although Tfh cells are critical for the generation of effective long-lived protective antibody responses, overrepresentation of Tfh cells is associated with systemic autoimmunity by producing pathogenic autoantibodies both in mouse and human studies [24–27]. The expansion of circulation Tfh cells was been found in several autoimmune diseases like systemic lupus erythematosus[24], rheumatoid arthritis[28] and primary biliary cirrhosis[29], etc. During the HBV infection, HBeAg to HBeAb seroconversion and further production of protective antibody HBsAb depend on the effective function of Tfh cells and B cells. In another hand, excessive activation of Tfh cells would contribute to the production of autoantibodies and lead to autoimmune diseases.It was reported that circulating CXCR5^+^CD4^+^T cells were expanded in patients with chronic hepatitis B[30, 31] and high frequency of circulating CXCR5^+^CD4^+^T cells were associated with HBeAg seroconversion through IL-21 production manner[31, 32].Our preliminary works have also shown the expansion of circulating Tfh cells and their associated molecules in patients with chronic HBV infection [33]. Correspondingly, the B cells in persistent HBV infection show an activated state and enhanced property to differentiate into plasma cells [11].However, the detailed profile and role of blood CXCR5^+^CD4^+^ T cells and B cells in patients with chronic HBV infection were not completely recovered.

In this study, we carefully investigated the phenotypes and functions of blood CXCR5^+^CD4^+^T cells and B cells in patients with chronic HBV infection. We also examined the autoimmune states of patients with chronic HBV infection and explored the correlation among blood CXCR5^+^CD4^+^T cells, B cells and HBV related autoimmune manifestation.

## Materials and Methods

### Patients

The study involved 85 outpatients or inpatients treated at the Second Affiliated Hospital of Chongqing Medical University between September 2011 and December 2014. The subjects included 42 asymptomatic HBV carriers (AsC), 43 patients with chronic hepatitis B (CHB), and 33 healthy controls (HC) (Table 1 and Fig A in S1 Data). In all patients, chronic HBV infection was diagnosed if HBsAg was positive and the serum HBV DNA was detectable for ≥6 months. Besides these 2 clinical parameters, AsC had normal liver function (alanine aminotransferase [ALT] level of <40 U/L, aspartate aminotransferase [AST] level of <30 U/L), patients with CHB had elevated aminotransferase. All serological markers for HBV were negative in HC. Patients were excluded if they were coinfected with other hepatitis viruses or HIV or had received antiviral or immunomodulatory treatment before blood sampling. Patients with primarybiliary cirrhosis, primary hepatocellular carcinoma were also excluded. These studies were conducted according to the Declaration of Helsinki guidelines, and were approved by the Ethical Committee of Second Affiliated Hospital of Chongqing Medical University. Written informed consent was obtained from all participants.

**Table 1:** Summary of patients and health controls in this study.

Parameters	HC	AsC	CHB
	n = 33	n = 42	n = 43
Age	33±12	38±13	36±12
Sex (M/F)	15/18	20/22	31/12
ALT (U/L)	<40	27.95±16.37	222.04±408.78
AST (U/L)	<30	26.26±8.23	180.37±407.12
HBV DNA (log copies/ml)	Negative	2.82±2.90	4.47±2.66

NOTE. Data are means±SDs.

### Flow Cytometry Analysis

Two-milliliter blood samples were taken out the plasma and red blood cells were lysed by NH4Cl lysis solution. Flow cytometry analysis was performed on 10^6^ cells per tube using the following fluorochrome-conjugated antibodies:anti-CD3–phycoerythrin (PE)–cyanine (CY) 7,anti-CD40L–PE, anti-CD19–phycoerythrin (PE)–cyanine (CY) 7, anti-CD40–PE, (eBioscience, San Diego, CA, USA), anti-CD4–fluoresceinisothiocyanate (FITC), anti-CXCR5–allophycocyanin (APC), anti-ICOS–PE, anti-PD1-PE, anti-IL-21R-PE, anti-CD27–fluoresceinisothiocyanate (FITC), anti-CD38–allophycocyanin (APC), anti-ICOSL–PE, anti-PDL1-PE (BD Company, San Jose, CA, USA). Isotype-matched control antibodies (BecktonDickinson, San Jose, USA) were used to correct nonspecific binding.Then the stained cells were analysed using a FACS Canto II cytometer and FACSDiva software (version4.1; Becton Dickinson).

### Isolation of Tfh Cells and B Cells

The peripheral blood mononuclear cells (PBMCs) from 20 milliliter blood samples were isolated on Ficoll-Hypaque gradients (Pharmacia Biotech). After CD4^+^ T lymphocytes were purified by CD4^+^ T cell isolation kit (Miltenyi Biotec), anti-CXCR5-Biotin (BD Biosciences) labeled CD4^+^ T cells were positively selected by binding to anti-Biotin microbeads (Miltenyi Biotec). For B cells isolation, anti-CD19-Biotin (BD Biosciences) labeled PBMC were positively selected by binding to anti-Biotin microbeads. Sorted cells with more than 90%purity were used.

### RT-PCR Analysis

Total cellular RNA was reverse-transcribed with PrimeScriptTM RT reagent Kit with gDNA Eraser (TaKaRa). cDNA was PCR amplified, electrophoresed, and visualized with ethidium bromide. For quantitative analysis, real-time RT-PCR was performed using SYBR Premix Ex TaqTMII (TaKaRa) and ABI 730 (Roche). Amplified products were confirmed to be of single bands over gel electrophoresis, and were normalized to the amount of GAPDH products.

### Tfh Cells and B Cells Coculture Assay

Sorted blood CD19^+^ B (5 × 104 per well) cells were cocultured with autologous CXCR5^+^CD4^+^ T cells or CXCR5^-^CD4^+^ T cells at a 1:1 ratio in the presence of 1 μg/mlof staphylococcal enterotoxin B (SEB) in 96-well U-bottom plates. Supernatants of culture cells were harvested on day 7 to analyze the total levels of Immunoglobulins (Igs), BLC and cytokines by ELISA.

### Cytokine and Ig Detection

The levels of plasma cytokines, the levels of cytokines and Igs in cells culture supernatants were measured using commercial human ELISA Kit (RayBiotech, Inc) in accordance with the manufacturer’s instructions.

### Autoantibody Detection

Presence of autoantibodies was detected by using commercial indirect immunofluorescence Kit or immunospot Kit (Euroimmune) in accordance with the manufacturer’s instructions.

### Statistical Analysis

All data were analyzed using SPSS 13.0 software (SPSS Inc, Chicago, IL, USA). Data were expressed as mean ± SD or mean ± SE. One-way ANOVA was used for comparisons between groups. The t test was used for two independent data. Pearson correlation test was used for correlation analysis. A two tailed P < 0.05 was considered statistically significant.

## Results

### The Phenotype of CXCR5^+^CD4^+^ T Cells Was Altered in Peripheral Blood of Patients with Chronic Hepatitis B Virus Infection

**Fig 1:**
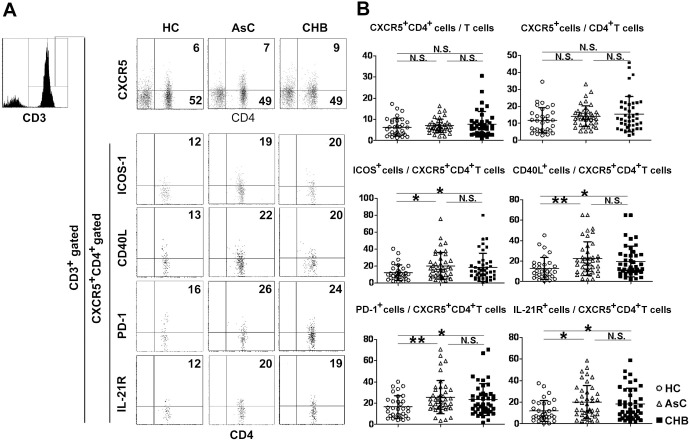
Fluorescence activating cell sorter (FACS) analysis of the frequency and phenotype of peripheral blood CXCR5^+^CD4^+^ T cells. (A) Peripheral blood from health control (HC, n = 33), asymptomatic HBV carriers (AsC, n = 42), and patients with chronic HBV infection (CHB, n = 43)were lysed by NH_4_Cl and cells were stained for CD3, CD4, CXCR5 and ICOS-1 / PD-1 / CD40L / IL-21R. The living mononuclearcells were gated initially on CD3^+^ T cells (upper panel) and then on CD4^+^CXCR5^+^T cells(lower four panels).Numbers indicate the frequency of cells within indicated areas.(B) The frequencies of total blood CXCR5^+^CD4^+^T cells and ICOS-1^+^/ PD-1^+^/ CD40L^+^/ IL-21R^+^CXCR5^+^CD4^+^T cells were compared between three groups. Means and standard deviations of cell frequency are shown. *, P < 0.05, **, P < 0.01, NS, not significant (P > 0.05).

To investigate whether the blood CXCR5^+^CD4^+^T cells are affected by the HBV infection, we examined the proportion and phenotype of CXCR5^+^CD4^+^T cells in peripheral blood from patients with chronic HBV infection and HC (Fig 1A and Fig B in S1 Data). The total frequency of blood CXCR5^+^CD4^+^ cells in T lymphocytes or CD4^+^ T lymphocytes was highest in CHB and lowest in HC, while the difference of which was not significant among HC, AsC, CHB groups(Fig 1B, upper panel). However, the frequency of CD40L expression cells in blood CXCR5^+^CD4^+^ T cells was significantly higher in either AsC or CHB patients, which was 22.47% and 19.78% respectively, than that in HC, which was 12.72%(Fig 1B, middle panel). The frequency of ICOS expression cells in blood CXCR5^+^CD4^+^ T cells was significantly higher in both AsC and CHB patients, which was 20.36% and 18.97% respectively, than that in HC, which was 12.20% (Fig 1B, middle panel). The expression of PD-1 and IL-21 receptors (IL-21R)in CXCR5^+^CD4^+^ T cells from both AsC and CHB patients was also significantly higher than that from HC(Fig 1B, lower panel). However, the expression of CD40L, ICOS, PD-1, IL-21R in blood CXCR5^+^CD4^+^ T cells were all comparable between AsC and CHB. We also found that the frequency and phenotype of blood CXCR5^+^CD4^+^ T cells were not different between HBeAg positive patients and HBeAg negative patients (S1 Fig).These results indicated that the phenotype of CXCR5^+^CD4^+^ T cells was affected by the HBV infection but not by the immune state of HBV infection.

### The Phenotype of Blood B Cells Was Moderately Altered in Peripheral Blood of Patients with Chronic Hepatitis B Virus Infection

**Fig 2:**
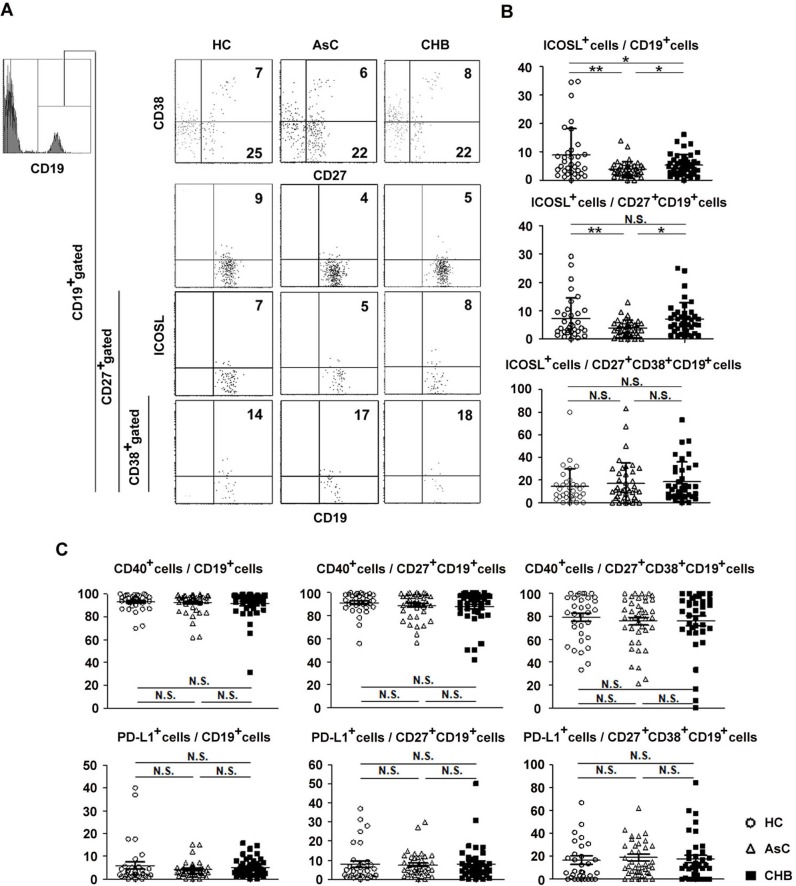
Fluorescence activating cell sorter (FACS) analysis of thefrequency and phenotype of peripheral blood CD19^+^ B cells. (A) Peripheral blood from HC (n = 33), AsC (n = 42) and CHB (n = 43)were lysed by NH_4_Cl and cells were stained for CD19, CD27, CD38 and ICOSL-1.The living mononuclearcells were gated initially on CD19^+^ B cells (upper two panels), then on CD27^+^ B cells(the third panels),and then on CD38^+^ B cells(lower panel).Numbers indicate the frequency ofcells within indicated areas.(B) The frequencies of ICOSL-1^+^ cells in total CD19^+^B, CD27^+^ memory B cells and CD27^+^CD38^+^ plasma cells were compared between three groups. (C)The frequenciesof PD-L1^+^ cells, CD40^+^ cells in total CD19^+^B, CD27^+^ memory B cells and CD27^+^CD38^+^ plasma cells were compared between three groups. Means and standard deviations of cell frequency are shown. *, P < 0.05, **, P < 0.01, NS, not significant (P > 0.05).

In order to find out whether blood B cells was affected by the HBV infection, we examined the frequency and phenotype of B cells in peripheral blood from patients with chronic HBV infection and HC (Fig 2A and Fig C in S1 Data). We found that the frequencies of total blood CD19^+^ B cells,CD27^+^CD19^+^ memory B cells and CD27^+^CD38^+^CD19^+^ plasma cells were comparable among AsC, CHB patients and HC (Fig 2A, upper panel). However, the frequency of ligand of ICOS (ICOSL) expression cells in total B cells from HC was significantly higher (8.87%) than that from AsC (3.86%) and CHB (5.34%)(Fig 2B, upper panel). ICOSL expression in memory B cells from HC was also significantly higher than that from AsC and CHB. Within the patients with HBV infection, the expression of ICOSL is significantly lower in total B cells and memory B cells from AsC than that from CHB (Fig 2B, middle panel). The expression of ICOSL in plasma cells was not different among AsC, CHB and HC (Fig 2B, lower panel). However, the expression of other molecules we examined, includingCD40 and PDL1in subsets of B cells was not different among HC, AsC and CHB patients (Fig 2C). These results indicated that the phenotype of B cells was mildly affected by the HBV infection.

### The Frequencies of Blood CXCR5^+^CD4^+^ T Cells and/or Frequencies of B Cells and Clinical Parameters in Patients with Chronic HBV Infection Were Correlated

**Fig 3:**
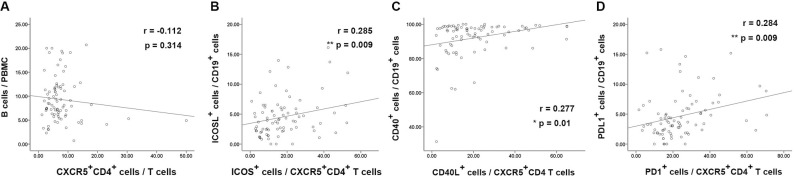
The correlation between frequencies of blood cell types in patients with chronic HBV infection. The correlation between the percentage of CXCR5^+^CD4^+^cells in total T cells and CD19^+^ B cells in PBMC. (B) The correlation between the percentage of ICOS^+^ cells in CXCR5^+^CD4^+^ T cells and ICOLSL^+^ cells in B cells. (C) The correlation between the percentage of CD40L^+^ cells in CXCR5^+^CD4^+^ T cells and CD40^+^ cells in B cells. (D) The correlation between the percentage of PD1^+^ cells in CXCR5^+^CD4^+^ T cells and PDL1^+^ cells in B cells in patients with chronic HBV infection (n = 85). *, P <0.05, 2-tailed, **, P < 0.01, 2-tailed.

We found that the frequency of total blood CXCR5^+^CD4^+^ T cells was not correlated with the frequency of total CD19^+^ B cells in patients with chronic HBV infection (P>0.05) (Fig 3A and S1 Table).Interestingly, the frequency of ICOS^+^CXCR5^+^CD4^+^ T was correlated positively with the frequency of ICOSL^+^ B cells, while the frequency of CD40L^+^CXCR5^+^CD4^+^ T was correlated positively with the frequency of CD40^+^ B cells in patients(Fig 3B and 3C and S1 Table). The frequency of PD1^+^CXCR5^+^CD4^+^ T was also correlated positively with the frequency of PDL1^+^ B cells in patients (Fig 3D and S1 Table). These results suggested the potential connection of blood CXCR5^+^CD4^+^ T and B cells in chronic HBV infection.

**Fig 4:**
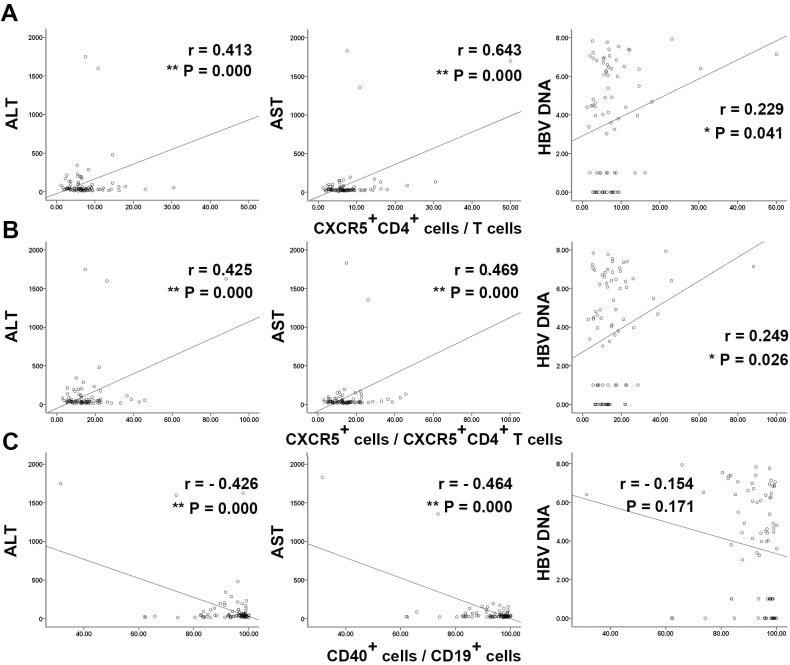
The correlation between frequencies of blood cells and clinical data in patients with chronic HBV infection. The correlation between the frequencies of CXCR5^+^CD4^+^cells in total T cells (A), CXCR5^+^cells in CXCR5^+^CD4^+^ T cells (B), CD40^+^cells in B cells (C) and serum levels of ALT (left), AST (middle), HBV-DNA (right) in patients with chronic HBV infection (n = 85). *, P <0.05, 2-tailed, **, P < 0.01, 2-tailed.

Next, we examined whether the blood CXCR5^+^CD4^+^ T cells and B cells were correlated with clinical parameters in chronic HBV infection. The analysis show that the frequencies of blood CXCR5^+^CD4^+^ T cells in total T cells and in CD4^+^ T cells, the frequency of blood plasma cells correlated positively with ALT and AST (Fig 4A and 4B and S2 Table). The frequency of blood CXCR5^+^CD4^+^ T cells in CD4^+^ T cells were also positively correlated with HBV load in blood (Fig 4A and 4B, right panel, and S2 Table). However, the frequency of CD40^+^cells in B cell populations was correlated negatively with ALT and AST, but not with HBV load in blood (Fig 4C and S2 Table). Collectively, in patients with chronic HBV infection, the frequencies of blood CXCR5^+^CD4^+^ T cell and B cells were associated with liver disease activity.

### Blood CXCR5^+^CD4^+^ T Cells from Patients with Chronic Hepatitis B Virus Infection Excessively Helped B Cells

**Fig 5:**
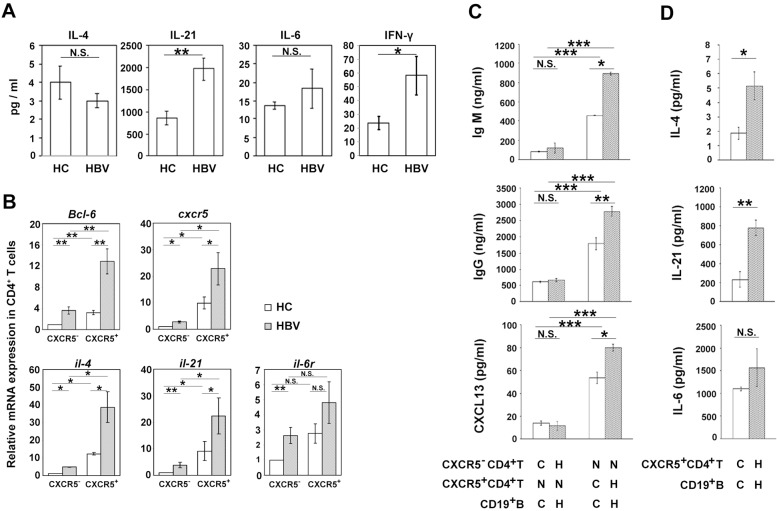
Function analysis of blood CXCR5^+^CD4^+^ T cells. (A)The concentration of serum Tfh-associated cytokines, including IL-4, IL-21, IL-6 and IFN-γ were performed by ELISA in health controls (HC, n = 20), patients with chronic HBV infection (HBV, n = 30). Means and standard errors of the concentration are shown.(B) Quantitative RT-PCR analysis of Bcl6, CXCR5, IL-4, IL-21, IL-6R expression in sorted CXCR5^-^CD4^+^ T cells and CXCR5^+^CD4^+^ T cells from health controls (HC, n = 6) and chronic HBV infected patients (HBV, n = 6) are shown. The amounts of examined genes were normalized to the amount of GAPDH, and those in CXCR5^-^CD4^+^ T cells from HC were arbitrarily set to 1. (C) IgM, IgG and CXCL13 (BLC) levels in the supernatant of B cells cocultured with the blood CXCR5^+^CD4^+^ T cells or CXCR5^-^CD4^+^ T cells from HC (C) and patients with chronic HBV infection (H) for 7 days. N, no cells. (D) IL-4, IL-21, IL-6 secretion from B cells cocultured with blood CXCR5^+^CD4^+^ T cell subsets from HC (C) and HBV infected patients (H) for 7 days. Bar graphs show means and standard errors. Representative results of three independent experiments are shown. *, P < 0.05, **, P < 0.01, ***, P < 0.001. NS, not significant.

Since the difference of frequency and phenotype of CXCR5^+^CD4^+^T cells were not significant between AsC and CHB, we didn’t separate the patients into these two groups in following analysis. When examine the serum concentration of several Tfh-associated cytokines, we found that the level of IL-21 was significantly increased in the patients with chronic HBV infection than that in HC, while the level of IL-6 and IL-4 was comparable between HC and patients (Fig 5A and Fig D in S1 Data).The IFN-γ was also significantly higher in patients with chronic HBV infection than that in HC (Fig 5A).

We then examined the expression of several function-related genes in CXCR5^+^CD4^+^ T cells and controlled CXCR5^-^CD4^+^ T cells which isolated from patients.CXCR5^+^CD4^+^ T cells express higher level of Tfh cell specific *Bcl-6*,*cxcr5* and cytokines*il-21* and *il-4* transcripts than CXCR5^-^CD4^+^ T cells both in patients and HC (Fig 5B and Fig E in S1 Data). The amount of *Bcl-6* expression in CXCR5^+^CD4^+^ T cells isolated from patients was about 3-fold of that from HC (P<0.05), while the amount of *cxcr5* expression in CXCR5^+^CD4^+^ T cells isolated from patients was about 2-fold of that from HC (P<0.05) (Fig 5B, upper panel). The expression of cytokine transcripts*il-4* and *il-21* was also higher in CXCR5^+^CD4^+^ T cells from patients than that from HC (Fig 5B, lower panel). The expression of *il-6r*, which is important to maintain the Tfh cells was not different from CXCR5^-^CD4^+^ T cells and CXCR5^+^CD4^+^ T cells either from patients or HC (Fig 5B, lower panel). These results indicated that the function of CXCR5^+^CD4^+^ T cells from patients with chronic HBV infection might be up-regulated versus HC.

Then we directly examined the helping capacity of blood CXCR5^+^CD4^+^ T cells to B cells in chronic HBV infection by cocultured these cells. To mimic the antigen-specific interaction between T and B cells, SEB was added to the cultures. Either from HC or patients, CXCR5^+^CD4^+^ T cells secret higher level of CXCL13 and induce B cells to produce much higher level of IgM, IgG than CXCR5^-^CD4^+^ T cells did (Fig 5C, Fig F in S1 Data).CXCR5^+^CD4^+^ T cells from patients could secret higher level of CXCL13,which is important to the interaction of Tfh and B cells, and induce B cells to secrete higher level of IgM and IgG than that from HC did (Fig 5C).CXCR5^+^CD4^+^ T cells from patients secreted larger amount of IL-21 and IL-4 that from HC (Fig 5D). However, CXCR5^+^CD4^+^ T cells from patients and HC secreted comparable level of IL-6 (Fig 5D, lower panel). These results indicated that the helping function of blood CXCR5^+^CD4^+^ T cells from patients with chronic HBV infection was magnified when compared with that from HC.

### Blood CXCR5^+^CD4^+^ T Cells from Chronic Hepatitis B Virus Infection Patients Were Positively Correlated with the Serum Autoantibodies

Finally, we examined the incidence of a series of non-organ-specificautoantibodies in patients with chronic HBV infection and whether it was correlated with the alteration of blood CXCR5^+^CD4^+^ T cells. Fifty-five persons (65%) among eighty-five patients had at least one positive autoantibody. However, there were only four persons (12%) among thirty-three health controls which had at least one positive autoantibody(Table 2, Fig G in S1 Data).In patients, the most common antoantibodies which present were antinuclear antibody (ANA) (25%), Anti SS-A antibody (ss-A) (21%),Anti SS-B antibody (ss-B) (14%) (Table 2).

**Table 2:** Non-organ-specific autoantibodies in patients with chronic hepatitis B and health controls.

Non-organ specific autoantibodies	HC	HBV
	(n = 33)	(n = 85)
Non autoantibody	29 (88%)	30 (35%)
At least one autoantibody	4 (12%)	55 (65%)
Antinuclear antibody (ANA)	3 (9%)	21 (25%)
Anti-mitochondrial antibody (AMA)	0 (0%)	2 (2%)
Anti smooth muscle antibody (ASMA)	0 (0%)	2 (2%)
Anti-liver-kidney microsomal antibody (LKM)	0 (0%)	0 (0%)
Anti-ribosomal P-protein autoantibody (nRNP/sm)	1 (3%)	9 (10%)
Anti Sm antibody (Sm)	2 (6%)	5 (6%)
Anti SS-A antibody (ss-A)	0 (0%)	15 (18%)
Anti SS-B antibody (ss-B)	0 (0%)	12 (14%)
Anti scl-70 antibody (scl-70)	0 (0%)	9 (11%)
Anti Jo-1 antibody (Jo-1)	0 (0%)	6 (7%)

NOTE. Data are numbers and percentage of the patients. HBV, patients with chronic hepatitis B virus infection

**Fig 6:**
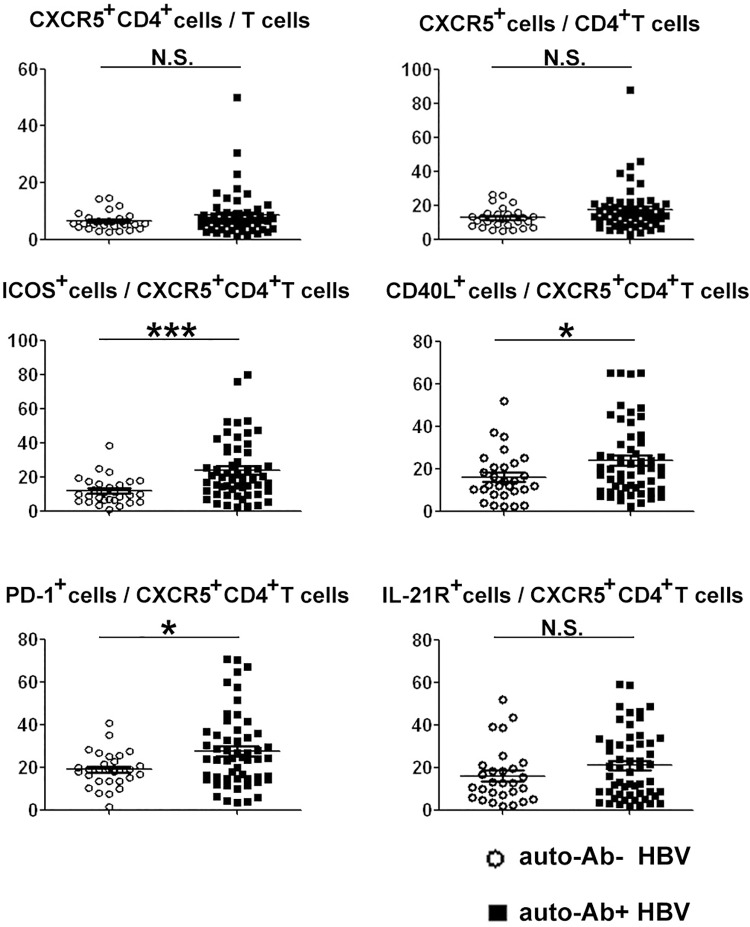
Flow cytometry analysis of the frequency and phenotype of blood CXCR5^+^CD4^+^ T cells from HBV patients with or without autoantibodies. The frequencies of total blood CXCR5^+^CD4^+^T cells and ICOS-1^+^/ PD-1^+^/ CD40L^+^/ IL-21R^+^CXCR5^+^CD4^+^T cells were compared between autoantibodies negative HBV patients (auto-Ab- HBV, n = 30), and autoantibodies positive HBV patients (auto-Ab^+^HBV, n = 55). Means and standard errors of cell frequency are shown. *, P < 0.05, ***, P < 0.001, NS, not significant.

When compared the phenotype of blood CXCR5^+^CD4^+^ T cells in the patients with antoantibodies and without autoantibodies, we found that the expression of PD-1, ICOS and CD40L but not IL-21R of blood CXCR5^+^CD4^+^, T cells from patients with autoantibodies was significantly higher than that from patients without autoantibodies (Fig 6).However, the level of ALT, AST, HBV DNA load and B cell phenotype in blood were not different between patients with or without autoantibodies (Data not show). These results indicated that the blood CXCR5^+^CD4^+^ T cells from patients with autoantibodies were more activated than that from patients without autoantibodies.

## Discussion

In this study, we found that the expression of function related molecules of blood CXCR5^+^CD4^+^ T cells were elevated in patients with chronic HBV infection. The expression of function related molecules in blood CXCR5^+^CD4^+^ T was positively correlated with the expression of corresponding ligands in B cells. The frequencies of blood CXCR5^+^CD4^+^ T and plasma cells were correlated positively to ALT and AST. Blood CXCR5^+^CD4^+^ T from patients secreted higher amount of IL-21, IL-4 and helped B cells to secret higher level of antibodies than that from HC did. Several antoantibodies were found in the patients. The expression of function related molecules in blood CXCR5^+^CD4^+^ T from HBV patients with autoantibodies was significantly higher than that from HBV patients without autoantibodies.

Several studies have described that the frequency of circulating CXCR5^+^CD4^+^ T cells was increased in chronic HBV infection [30, 32].However, our results show that the difference of the frequency of total blood CXCR5^+^CD4^+^ T cells was not significant among AsC, CHB patients and HC(Fig 1). This controversial could be explained by that the average age of the research aims[32] and the flow cytometry analysis gate for blood CXCR5^+^CD4^+^ T cells[30] were different between our research and the reported ones. We defined blood Tfh cells as CD3^+^CXCR5^+^CD4^+^ PBMC as while the reported ones defined them as CXCR5^+^CD4^+^ PBMC [30]. Tfh cells express a unique combination of effecter molecules that are critical for their development and function, including high levels of the surface receptors ICOS[21], CD40L, PD-1[23], IL-21 and the receptor IL-21R[19, 20]. CD40L-CD40 signal promotes B cells for proliferation, while IL-21 enhances the differentiation and immunoglobulin secreting of CD40L-stimulated B cells. Engaging ICOS on CD4^+^ T cells induces the production of cytokines IL-21.PD-1 is expected to provide an inhibitory signal to Tfh cells. On the other hand, PD-1 also provides direct inductive signals from GC Tfh to B cells. We found that the expression of ICOS, CD40L, IL-21R, even PD-1 in blood CXCR5^+^CD4^+^ T cells were significantly elevated in the patients with chronic HBV infection, but was comparable between AsC and CHB(Fig 1). These results indicate that high expression of function related molecules in blood CD4^+^CXCR5^+^ T cells was affected by the HBV infection but not by the immune-active or immune-tolerant state of chronic HBV infection. The expression of *Bcl-6*,*cxcr5* and cytokine genes in the blood CD4^+^CXCR5^+^ T cells from patients were also increased versus that from HC(Fig 5B).The amount of serum IL-21 was higher in patient than that in HC (Fig 5A). All these results indicated that the blood CXCR5^+^CD4^+^ T cells in chronic HBV infection might be over activated.

When directly examine the helping function, we found that blood CXCR5^+^CD4^+^ T cells from patients with chronic HBV infection have increased capacity to secret more IL-21, IL-4 and help B cells for more Igs secreting than that from HC did (Fig 5C and 5D). These findings directly verified that the blood CXCR5^+^CD4^+^ T cells in chronic HBV infection were over activated and have enhanced capacity to help B cells for antibody production. Excessive generation of Tfh cells likely contributes to the production of pathogenic autoantibodies in several human autoimmune conditions[24–27]. It has been reported that in persistent virus infection, including HBV and HCV, series of non-organ-specific autoantibodies were produced and affected a variety of organs or tissues besides liver lesions[4–7].We did find that series of non-organ-specific autoantibodies including ANA, ss-A, ss-B, scl-70, nRNP/sm, etc were positive in 65% patients with chronic HBV infection in Chinese population (Table 2). The expression of function-related molecules in blood CXCR5^+^CD4^+^ T cells in HBV patients with autoantibodies were significantly higher than that in patients without autoantibodies (Fig 6). Our results exactly suggested that the generation of autoantibodies might be caused by the over activation of Tfh cells in chronic HBV infection. Although the incidence of autoimmune diseases in patients with chronic hepatitis B was not investigated in this research, it could be presumed that the autoimmune manifestations might be associated with the augmented function of blood CXCR5^+^CD4^+^ T cells. However, the molecular mechanisms that how blood CXCR5^+^CD4^+^ T cells were activated by HBV infection needs to further be investigated.

Research of B cells in HBV infection showed that although the frequency of blood CD19^+^ cells were not affected, CD27^+^ memory B cells were enhanced to differentiate into plasma cells compared with controls [11]. In our research, we find that the frequency of total blood B cells, memory B cells and plasma cells in patients with chronic HBV infection were comparable with that in HC. When examined the molecules which are important for the Tfh-B cell interaction, we found that the expression of CD40 and PD-L1 in B cells were not affected by HBV infection, while the expression of ICOSL was decreased in CHB patients and even lower in AsC when compared with that from HC (Fig 2).It was interesting that blood B cells from patients with chronic HBV infection could produce more Igs when co-cultured with CXCR5^+^CD4^+^ T cells than that from HC did (Fig 5C).These results suggested, although the alteration of B cells phenotype was moderate, blood B cells were ready to produce elevated level of antibodies when helped by Tfh cells in HBV infection.

The expression of function related molecules, including ICOS, CD40L, PD-1 in the blood CD4^+^CXCR5^+^ T cells were positively correlated to the expression of corresponding ligands in the B cells respectively (Fig 3 and S1 Table).These findings indicated a connection of blood CXCR5^+^CD4^+^ T cells and B cells in patient with chronic HBV infection, supporting the notion that the interaction of Tfh cells and B cells promote the survival and function bilaterally. It has been reported that the frequency of CXCR5^+^CD4^+^ T cells was positively correlated to serum AST [30].We also found the frequency of blood CXCR5^+^CD4^+^ T cells in either T cells or CD4^+^ T cells and the frequency of blood CD27^+^ memory B cells were positively correlated with ALT, AST, while the CD40 expression in subsets of B cells was negatively correlated with ALT and AST (Fig 4 and S2 Table). These findings suggested that these cells somehow related to the inflammatory in the liver in chronic HBV infection.

Finally, it was also reported that the elevated frequency of CXCR5^+^CD4^+^ T cells was related to the HBeAg to HBeAb seroconversion [32].However, we didn’t find that the frequency and phenotype of blood CXCR5^+^CD4^+^ T cells were different between HBeAg positive patients and HBeAg negative patients which were HBeAb positive (S1 Fig), indicating the function augmenting of Tfh cells and B cells in chronic HBV infection was not HBV antigen specific and didn’t profit the production of HBeAb or protective antibody HBsAb. It was suggested that the generation of HBeAb or HBsAb might depend on the activation of HBV antigen specific Tfh cells.

## Conclusion

In conclusion, the present results indicate that the blood CXCR5^+^CD4^+^T cells from patients with chronic HBV infection were over activated and have augmented capacity to help B cells for antibody secreting. A series of non-organ-specific autoantibodies did be positive in chronic HBV infection and were correlated with the activation state of blood CXCR5^+^CD4^+^ T cells. The frequency of blood CXCR5^+^CD4^+^ T cells and memory B cells were also correlated with liver inflammatory in HBV infection. Our research imply a novel clue that the production of autoantibodies might be due to the over activation of Tfh cells in chronic HBV infection.We could regulate the function of Tfh cells as therapy target to control the liver inflammatory and extrahepatic autoimmune-related lesions in chronic HBV infection.

## Supporting Information

S1 Data Raw datas of each figures. (A) Summary of patients and health controls. (B) Raw data of Tfh cells and phenotype. (C)Raw data of B cells and phenotype. (D)Cytokines expression in the serum. (E) Normalized expression of genes by ct value of Real-time PCR. (F) Ig and chemokine secretion in the culture cells (G)Autoantibodies expression in patients.
(PDF)

S1 Fig Fluorescence activating cell sorter (FACS) analysis of the frequency and phenotype of peripheral blood CXCR5^+^CD4^+^ T cells. The frequencies of total blood CXCR5^+^CD4^+^T cells and ICOS-1^+^/ PD-1^+^/ CD40L^+^/IL-21R^+^CXCR5^+^CD4^+^T cells were compared among health control (HC, n = 33), HBeAg positive patients with chronic HBV infection (HBeAg ^+^, n = 29), and HBeAg negative patients with chronic HBV infection (HBeAg -, n = 56). Means and standard deviations of cell frequency are shown. *, P < 0.05, **, P < 0.01, NS, not significant (P > 0.05) (Student's t-test).
(PDF)

S1 Table Pearson correlation coefficients for frequencies of blood cell types in patients with chronic HBV infection. (PDF)

S2 Table Pearson correlation coefficients for frequencies of blood cells and clinical data in patients with chronic HBV infection. (PDF)
