# Supplementary material for: Production of Autoantibodies in Chronic Hepatitis B Virus Infection Is Associated with the Augmented Function of Blood CXCR5+CD4+ T Cells
Source: PLoS One. 2016 Sep 9;11(9):e0162241. doi: 10.1371/journal.pone.0162241 (PMC5017876; doi:10.1371/journal.pone.0162241)
Supplement: S1 Data — (A) Summary of patients and health controls. (B) Raw data of Tfh cells and phenotype. (C)Raw data of B cells and phenotype. (D)Cytokines expression in the serum. (E) Normalized expression of genes by ct value of Real-time PCR. (F) Ig and chemokine secretion in the culture cells (G)Autoantibodies expression in patients. (PDF) [file pone.0162241.s001.pdf]

A.Summary of patients and health controls

No	Group	Sex	Age	HBsAg	HBsAb	HBeAg	HBeAb	HBcAb	HBV-DNA log copies/m	ALT U/L	AST U/L
1	CHB	M	43	+	−	−	+	+	1	65	130
2	CHB	M	26	+	−	−	+	+	7.139	1625	1698
3	CHB	F	16	+	−	+	−	+	6.06	174	84
4	CHB	M	27	+	−	−	+	+	6.514	1598	1355
5	CHB	M	29	+	−	−	+	+	6.408	1748	1831
6	CHB	M	47	+	−	+	+	+	7.938	31	86
7	CHB	M	35	+	−	−	+	+	6.371	479	171
8	CHB	M	31	+	−	−	+	+	6.409	54	133
9	CHB	M	16	+	−	+	−	+	6.853	66	39
10	CHB	M	25	+	−	−	+	+	0	116	53
11	CHB	M	44	+	−	−	+	+	4.466	170	79
12	CHB	M	11	+	−	+	−	+	6.082	91	65
13	CHB	F	37	+	−	+	−	+	6.604	196	159
14	CHB	M	39	+	−	−	+	+	0	51	39
15	CHB	F	42	+	−	−	+	+	4.676	67	32
16	CHB	F	60	+	−	+	−	+	5.494	112	67
17	CHB	M	27	+	−	+	−	+	6.705	342	149
18	CHB	F	35	+	−	−	−	+	0	44	25
19	CHB	M	41	+	−	−	+	+	0	46	28
20	CHB	F	56	+	−	−	+	+	4.406	82	57
21	CHB	M	34	+	−	+	−	+	4.498	214	98
22	CHB	M	48	+	−	−	+	+	3.38	52	34
23	CHB	F	31	+	−	−	+	+	0	88	66
24	CHB	F	44	+	−	−	+	+	0	44	40
25	CHB	M	34	+	−	+	+	+	6.896	231	150
26	CHB	F	19	+	−	+	−	+	6.826	132	102
27	CHB	M	22	+	−	+	−	+	6.475	62	33
28	CHB	M	62	+	−	+	−	+	6	47	29
29	CHB	M	38	+	−	+	−	+	7.363	47	33
30	CHB	M	41	+	−	−	+	+	4.812	45	38
31	CHB	M	45	+	−	−	+	+	1	47	32
32	CHB	M	26	+	−	−	−	+	7.23	42	32
33	CHB	F	57	+	−	−	+	+	3.257	286	194
34	CHB	M	54	+	−	−	+	+	3.965	61	33
35	CHB	F	34	+	−	−	+	+	0	39	41
36	CHB	M	44	+	−	−	+	+	0	41	32
37	CHB	M	47	+	−	−	+	+	3.804	93	68
38	CHB	M	29						6.947	47	34
39	CHB	M	22	+	−	−	+	+	4.426	136	73
40	CHB	M	39	+	−	−	+	+	6.225	195	76
41	CHB	M	39	+	−	−	+	+	6.225	195	76
42	CHB	M	38	+	−	−	+	+	3.021	45	41
43	CHB	F	28	+	−	+	−	+	6.981	202	121
44	AsC	F	77	+	−	−	+	+	0	18	42
45	AsC	M	45	+	−	−	+	+	1	35	40
46	AsC	M	22	+	−	+	−	+	7.064	14	22
47	AsC	M	29	+	−	+	−	+	7.406	34	18
48	AsC	F	22	+	−	−	+	+	0	20	16
49	AsC	M	25	+	−	−	+	+	0	116	53
50	AsC	M	44	+	−	−	+	+	3.969	30	25
51	AsC	F	33	+	−	−	+	+	4.127	16	22
52	AsC	F	21	+	−	+	−	+	7.056	25	21
53	AsC	F	44	+	−	−	+	+	1	17	35
54	AsC	F	35	+	−	+	−	+	6.276	40	39
55	AsC	M	39	+	−	−	+	+	0	14	19

56	AsC	M	48	+	-	-	+	+	1	40	31
57	AsC	F	56						3.598	12	27
58	AsC	M	28	+	-	+	-	+	7.764	36	28
59	AsC	M	33	+	-	-	+	+	0	11	16
60	AsC	F	54	+	-	-	+	+	0	20	27
61	AsC	M	27	+	-	-	+	+	4.029	40	12
62	AsC	M	32	+	-	-	+	+	4.404	28	23
63	AsC	F	28	+	-	+	-	+	7.835	35	38
64	AsC	M	58	＋	－	－	＋	＋	1	20	29
65	AsC	F	59	＋	－	－	＋	＋	0	14	22
66	AsC	M	32	＋	－	－	＋	＋	4.92	39.1	36
67	AsC	F	25	＋	－	＋	－	＋	7.537	16	22
68	AsC	M	41	＋	－	－	＋	＋	0	24	19
69	AsC	F	36	＋	－	－	＋	＋	1	34	25
70	AsC	F	56	＋	－	－	＋	＋	0	23	26
71	AsC	F	36	＋	－	－	－	＋	0	19	20
72	AsC	F	37	＋	－	－	＋	＋	0	23	26
73	AsC	F	58	＋	－	－	＋	＋	7.4003	31	31
74	AsC	F	41	＋	－	＋	－	＋	7.404	23	21
75	AsC	M	50	＋	－	－	＋	＋	0	31	29
76	AsC	F	43	＋	－	－	＋	＋	0	31	25
77	AsC	F	41	＋	－	－	＋	＋	1	15	20
78	AsC	M	33	＋	－				1	27	24
79	AsC	M	57	＋	－				1	26	15
80	AsC	M	30	＋	－	＋	＋	＋	4.898	33	24
81	AsC	M	29	＋	－	－	－	＋	2.91	26	25
82	AsC	F	22	＋	－				4.622	39	34
83	AsC	M	27	＋	－	－	＋	＋	1	33	32
84	AsC	F	20	＋	－	＋	－	＋	5.39	26	19
85	AsC	F	29	＋	－	－	＋	＋	1	20	25
86	HC	M	34								
87	HC	F	25								
88	HC	F	24								
89	HC	F	25								
90	HC	F	24								
91	HC	M	35								
92	HC	M	34								
93	HC	F	33								
94	HC	F	25								
95	HC	F	24								
96	HC	M	26								
97	HC	F	26								
98	HC	M	26								
99	HC	M	64								
100	HC	F	60								
101	HC	M	32								
102	HC	F	27								
103	HC	M	27								
104	HC	F	25								
105	HC	M	27								
106	HC	F	24								
107	HC	F	26								
108	HC	M	26								
109	HC	M	24								
110	HC	F	44								
111	HC	M	58								
112	HC	F	62								
113	HC	M	38								

114	HC	F	35
115	HC	F	40
116	HC	M	45
117	HC	F	44
118	HC	M	29

B. Raw data of Tfh cells and phenotype

No	Group	CD4 in T	CXCR5CD4 in T	CXCR5CD4 in CD4	ICOS in T	ICOS in CD4	ICOS in Tfh	PD1 in T	PD1 in CD4	PD1 in Tfh	CD40L in T	CD40L in CD4	CD40L in Tfh	IL21R in T	IL21R in CD4	IL21R in Tfh
1	CHB	72.7	16.2	22.696	6.7	9.215	14.1	6.2	8.933	14.4	7.6	11.515	20.7	9.7	14.119	20.5
2	CHB	56.8	50	88.028	14	24.647	24.8	11.8	20.774	23.7	21.3	37.5	45.5	12.3	21.654	31.2
3	CHB	29.2	6.6	22.602	12.4	42.465	51.8	18	61.643	67	16.7	57.191	64.8	12.8	43.835	42.8
4	CHB	41.5	10.8	26.024	0.9	2.168	3.4	3.4	8.192	8	1.1	2.65	2.7	2.1	5.06	6
5	CHB	50.7	7.5	14.792	1.8	3.55	5.4	0.2	0.394	1.6	0.7	1.38	2.2	1.6	3.155	9.8
6	CHB	53.9	23.1	42.857	6.2	11.502	13.8	11.2	20.779	26.8	7.7	14.285	20.4	9.9	18.367	20.6
7	CHB	66.2	14.6	22.054	3	4.531	6.2	3.9	5.891	9.9	2.3	3.474	7.6	2	3.021	3.7
8	CHB	66.7	30.5	45.727	28.3	42.428	46.4	24.5	36.731	37.5	14.8	22.188	25.5	9.4	14.092	16
9	CHB	57.1	8.8	15.411	6.5	11.383	16.3	3.8	6.655	11.6	4.2	7.355	11.2	1.5	2.626	5.1
10	CHB	45.8	5.7	12.445	4	8.733	11.9	4.6	10.043	14.8	2.8	6.113	16.9	2.2	4.803	8.8
11	CHB	27.3	2.3	9.523	14.9	54.578	52.4	17	62.271	70.4	10.2	37.362	43.5	16.4	60.073	58.9
12	CHB	43.7	5.6	12.814	39.2	89.703	80	9.8	22.425	36.7	6.2	14.187	27	7	16.018	40.2
13	CHB	57.8	6.5	11.245	4.8	8.304	19.6	11.2	19.377	33.8	5.2	8.996	26.4	7.7	13.321	34.2
14	CHB	42.4	9.2	21.8	3.2	7.547	15.2	7.5	17.688	26.7	3.3	7.783	18.9	3.6	8.49	13.3
15	CHB	46.3	17.9	38.66	11.3	24.406	27	14.9	32.181	42	13.1	28.293	32.2	6.5	14.038	19.8
16	CHB	40	14.6	36.5	14.5	36.25	39.1	14.4	36	44.6	11.7	29.25	35.7	10.5	26.25	31.6
17	CHB	53.5	5.4	10.093	5.4	10.093	17.9	11.2	20.935	28.4	3.5	6.542	10.1	3.3	6.168	15.5
18	CHB	54.5	7.7	14.128	7.9	14.495	23.3	8.8	16.147	16.8	5.9	10.825	29.1	2	3.669	10.9
19	CHB	49	5.7	11.632	3	6.122	9.7	2.3	4.694	7.4	1.6	3.265	7.7	7.2	14.693	18.2
20	CHB	40.7	1.2	2.948	5.1	12.53	14.9	12.6	30.958	45.2	4	9.828	12.2	8.1	19.901	35.2
21	CHB	48.8	2.7	5.532	2.8	5.982	9.2	6.3	12.91	18.9	3.7	7.613	10.4	2	4.098	8.5
22	CHB	46.8	1.7	3.624	2.3	4.912	16.7	7	14.957	24.1	3.9	8.333	14.3	3.8	8.119	7.7
23	CHB	59.5	3.7	6.218	3.5	5.824	0.8	11.3	18.992	17.7	3.7	6.218	9.4	1.9	3.193	3.3
24	CHB	28.2	2.7	9.574	13.3	47.163	43.2	10.7	37.943	41.6	16.8	59.574	65	8.3	29.432	31.5
25	CHB	28.3	5.5	19.434	2.4	8.48	9.9	1.2	4.074	9.7	3.3	10.091	10.3	1	3.105	5.4
26	CHB	32.2	3.7	11.49	1.2	3.738	4.4	1.7	5.06	11.6	3.1	8.635	8.2	2.7	6.474	4.8
27	CHB	39	6.7	17.179	3	7.692	9.2	2.5	6.41	12	2.8	6.65	5	3.4	8.353	11.7
28	CHB	47.2	9.1	19.279	1.2	2.553	2.7	2.1	4.449	4	5.7	11.875	11.8	1.2	2.58	2.1
29	CHB	57.9	12.2	21.07	4.8	8.556	8.7	10.5	19.373	27.8	4.8	8.377	8.1	2.2	4.036	7.2
30	CHB	41.3	2.8	6.779	1.7	4.106	2.9	7.9	18.203	19.1	4.4	11.139	16.4	0.7	2.108	4.5
31	CHB	37.4	8.3	22.192	4.3	11.497	13	6.1	15.327	14.8	2.1	5.236	3.9	1.9	4.051	3
32	CHB	56.2	2.8	4.982	3.2	5.694	7	3.2	5.665	15.1	1.6	2.807	11.8	1.5	2.564	5
33	CHB	60.3	8.3	13.764	9.7	16.086	25.1	13.5	22.388	24.4	5.4	8.955	34.9	9.1	15.091	48.7
34	CHB	44.5	3.4	7.64	5.8	13.033	21.4	5.7	12.809	17.7	5.3	11.91	41.8	5.3	11.91	45.8
35	CHB	50.2	3.4	6.772	14.5	28.884	42.3	14.3	28.486	29.3	8.9	17.729	25.4	9	17.928	23
36	CHB	45.9	3.1	6.753	5.3	11.546	13.8	11.5	25.054	25.2	11.5	25.054	25.2	16.5	35.947	38.6
37	CHB	50.6	9.3	18.379	3	5.928	10.7	5.6	11.067	13.5	5.4	10.671	11.7	3.3	6.521	16.8
38	CHB	45.5	2.8	6.153	5.5	12.087	20.6	6.3	13.846	22.9	4.4	9.67	17	4.7	10.329	26.3
39	CHB	59.6	3.3	5.536	5.2	8.724	18.5	11.4	19.128	28.6	5.4	9.06	9.6	4.7	7.885	18
40	CHB	70.3	6.2	8.819	4.3	6.116	6.2	6.2	8.696	10.2	3.4	4.836	12.3	2.4	3.413	10.8
41	CHB	70.3	6.2	8.819	4.3	6.116	6.2	6.2	8.696	10.2	3.4	4.836	12.3	2.4	3.413	10.8
42	CHB	61	6.3	10.327	3.1	5.082	5.2	7.2	11.803	13.5	1.4	2.295	4	0.8	1.311	2.1
43	CHB	43.6	5.6	12.844	4.7	10.78	12.1	6.5	14.908	19.2	4.2	9.633	14.2	3.3	7.568	6.9
44	AsC	49.3	16.3	33.062	26.5	44.463	47.4	19.7	35.559	57.6	31.7	55.034	65.2	30	50.847	48.7
45	AsC	44.7	5.7	12.751	23.1	51.4476	52.9	19.5	45.667	51.3	19.3	64.3	46.618	16	34.934	46.1
46	AsC	40.2	7	19.073	26.7	72.752	75.7	27.8	69.154	70.7	19.8	50	48.5	20.8	47.926	58.6
47	AsC	44.7	6.9	15.436	14.7	32.885	36.4	7.2	16.107	23.9	9.8	21.923	22.3	11.3	25.279	33.7
48	AsC	38.1	5.1	13.385	4.4	11.548	15.4	4.1	10.761	18.9	3.1	8.136	14.2	3.3	8.661	21.1

49	AsC	45.8	5.7	12.445	4	8.733	11.9	4.6	10.043	14.8	2.8	6.113	16.9	2.2	4.803	8.8
50	AsC	54.1	14.2	26.247	4.4	8.133	10	7.2	13.308	19.7	5.9	10.905	20.8	17.7	32.717	43.3
51	AsC	44.4	6.7	15.09	5.8	13.063	20.5	11.7	26.351	35.1	4.6	10.36	23.3	7.6	17.117	27.8
52	AsC	37.6	2	5.319	13.6	36.17	45.7	16.4	43.617	59.7	14.5	38.563	50	15.4	40.957	43.3
53	AsC	47.7	13.6	28.511	8.5	17.819	18.1	10.7	22.431	30.3	7.2	15.094	20.5	8.7	18.238	30.8
54	AsC	49.9	6.1	12.224	2.2	4.408	5.7	7.2	14.428	20.6	5.4	10.821	24.9	2.2	4.408	10.2
55	AsC	51.9	6.4	12.331	8.4	16.185	18.1	6.4	12.331	23.3	0.7	1.348	5.7	2.6	5.009	21.4
56	AsC	61.4	4.6	7.491	4.7	7.654	14.9	10.4	16.938	19.7	10.1	16.449	31.3	4.7	7.654	8.8
57	AsC	44.7	7.4	16.554	2.3	5.145	6.9	6.8	15.213	23.6	2.1	4.697	15	2.6	5.816	12.2
58	AsC	48.9	6.4	13.087	6.1	12.474	15.7	8.5	17.382	16.3	7.9	16.155	22.6	2.7	5.521	12.8
59	AsC	54.9	5.6	10.2	5	9.107	17.4	4.6	8.379	13.5	4.8	8.743	20.7	2.1	3.825	12.9
60	AsC	34	4.3	12.647	3.5	10.294	25	4.3	12.647	25.3	3.5	10.294	34.9	1.6	4.705	12.6
61	AsC	36.6	5.3	14.808	5.7	15.573	22.9	5.1	13.934	22.8	1.4	3.825	11.7	2.3	6.284	22.3
62	AsC	53.3	11.2	21.013	5.4	10.131	14.8	4.3	8.067	15.7	3.6	6.754	17.3	3.5	6.566	13
63	AsC	47.6	2.6	5.462	17	35.714	34.1	15	31.513	32.6	8.2	17.2226	19.8	12.2	24.596	29.2
64	AsC	50	8.7	17.4	17.8	35.6	24.5	16.9	32.5	28	1.8	5.07	7.1	2.5	7.309	8.5
65	AsC	42.8	9.4	21.962	2.1	4.895	3.5	2.5	5.252	4.2	1.4	3.508	2.3	1.9	5.08	3.6
66	AsC	44.6	7.6	17.04	1.4	3.153	3.2	1.2	2.778	3.4	5	11.16	8.9	1.1	2.612	2.5
67	AsC	61.7	9.4	15.235	7.3	11.831	15.3	3	4.918	5.9	8.8	15.521	14.2	2	3.527	2
68	AsC	55.3	7.4	13.381	15.3	26.333	26.5	5.8	9.699	12.9	5	8.818	12.7	4.7	7.679	12.3
69	AsC	38.4	6.5	16.927	5.4	14.248	9.8	6.3	18.314	24.5	23.4	46.893	65	1.5	3.318	6.7
70	AsC	63.1	8.5	13.47	4	7.533	9.2	8	12.308	15.6	2.2	3.571	6.7	2	3.081	3.2
71	AsC	59.4	7.9	13.3	1.5	2.523	2.4	2.4	3.84	6.3	3.4	5.28	9.5	3.1	4.73	7.3
72	AsC	40.1	3.6	8.977	1.8	4.5	6.7	2.5	5.995	16.1	2.8	7.197	11.2	2	5.089	11.6
73	AsC	66.8	10.8	16.167	2.8	4.197	5.3	5.9	8.298	14.3	1.6	2.335	6.5	1.1	1.551	3.1
74	AsC	53.2	12	22.944	4.1	7.7068	11.2	11.8	22.18	26.6	2.3	4.323	10.4	5.1	9.586	18.1
75	AsC	71.8	7.6	10.584	10.2	14.206	19.4	18.4	25.627	35	17.4	24.233	51.9	8.9	12.395	52
76	AsC	66.9	5.2	7.772	15.4	23.019	38.1	25.5	38.117	40.5	17.8	26.606	37	9.4	14.05	39.2
77	AsC	55.5	4.6	8.288	5	9.009	8	10.1	18.189	21.3	4.4	79.279	16.7	4.4	79.279	25.3
78	AsC	68.5	3.7	5.401	8.3	12.116	17.2	10.3	15.036	27.5	8.8	12.846	28.9	3.7	5.401	19.6
79	AsC	42.4	2	7.716	16.7	39.386	34.7	24.4	57.547	64.8	14.9	35.141	44.8	5.5	12.971	24
80	AsC	37.4	5.882	9	9	24.064	37.5	9.6	23.881	32.4	3.7	8.222	23.8	4.9	11.893	23
81	AsC	53	8.2	15.471	4.9	9.245	9	6.7	12.665	17.8	2.7	5.222	18.3	1.9	3.682	8.1
82	AsC	44.3	3.3	7.449	9.3	20.9932	28.9	11.9	26.862	35.7	3.8	8.577	23.7	6.6	14.898	33.3
83	AsC	44.3	4.6	10.383	8.9	20.09	22.3	6.1	13.77	15.3	1.9	4.288	7.3	2.2	4.966	4.6
84	AsC	41.6	6.6	15.865	1.8	4.327	5	3	7.212	16.7	0.8	1.923	2.7	5.8	14.02	21.6
85	AsC	47.5	6.2	13.052	2.7	5.684	8.1	3.2	6.737	18.8	0.9	1.894	2.1	0.4	0.842	1.9
86	HC	52.3	10.2	19.502	2.3	4.397	5.4	2.5	4.78	6.6	14.6	6.118	13.7	4	7.648	18.7
87	HC	51.1	9.4	18.395	1	1.956	1.7	2.1	4.109	4.2	1.9	3.7181996	3.7	0.8	1.565	2.6
88	HC	50.4	6.3	12.5	6.3	12.5	8.4	1.3	2.579	7.9	0.2	0.3968	1	0.1	0.198	0.4
89	HC	44.5	1.3	2.921	4.6	10.337	35.4	4.1	9.213	5.4	4.8	10.786	45.2	2.8	9.12	19.1
90	HC	30.7	3.257	12.052	4.9	15.961	23.5	6.7	21.824	33.3	4.1	13.355	18.2	4.6	14.983	7.7
91	HC	39	8.6	22.051	5.1	13.076	20.3	5.7	14.615	15.6	2.6	6.666	14.9	2.5	6.41	14.1
92	HC	40.6	14	34.482	9.9	23.349	21.2	13.2	32.512	36	11.8	27.339	36.4	3.9	9.605	11.8
93	HC	70.1	10	14.265	2.4	3.423	8.9	2.2	3.138	4	1.5	2.139	7.2	1.4	1.997	6.1
94	HC	69.2	2.9	4.19	0	4.768	12	5.1	7.37	10	3.9	5.635	9.8	3.6	5.202	6
95	HC	45	1.7	3.777	4.2	9.333	11	7.3	16.222	21.7	5.9	13.11	15.2	3	6.666	11
96	HC	59.7	6.8	11.39	1.5	2.521	6.3	1.4	2.345	8.7	0.2	0.335	1.7	0.4	0.67	3.1
97	HC	56.6	11.8	20.848	1.3	2.296	4	2.7	4.77	8.2	2.2	3.886	10.4	1.8	3.18	6.8
98	HC	52.4	8.6	16.412	1.3	2.48	4.1	1.2	2.844	5.5	1.1	2.165	5.2	0.7	1.383	2.3
99	HC	59.3	9.5	16.02	2.8	4.721	6.9	3.1	5.228	9.3	2.1	3.541	5.3	2.9	4.89	7
100	HC	72.4	15.1	20.856	6.6	9.103	11.2	6.9	9.426	14.8	2.7	3.818	6.1	3	4.444	6.7
101	HC	44.3	6.3	14.221	3.4	7.674	10.2	4.4	10.092	12.2	2.4	6	8.4	3.1	8.179	8.1
102	HC	52.8	7.4	14.015	3.4	6.439	10.5	3.4	6.489	15.8	1.3	2.462	2.6	1.2	2.234	3.3
103	HC	44.8	5.3	11.83	2.9	6.487	7.5	5.9	11.943	12.4	3.7	8.149	9.5	2.4	8.856	9
104	HC	49.7	2.4	4.828	4	8.048	14.8	5.6	11.268	15.4	3.7	7.444	16.5	3.4	6.841	20.4
105	HC	56	3.4	6.071	6.7	11.964	11.4	8.1	14.464	21.7	7.1	12.678	18.5	2.9	4.915	12
106	HC	34.4	2	5.813	11.6	33.72	17.4	8.2	23.837	26.9	8	23.255	24.5	3.9	11.337	25

107	HC	37.4	2.4	6.417	6.4	17.1123	17.2	8.3	22.193	31	3.8	10.16	17.4	2	5.347	22.2
108	HC	41.1	1.7	4.136	13.4	32.603	40.5	12.3	29.927	26.5	10.9	26.52	33.3	5.7	13.868	13.8
109	HC	52.4	1.8	3.435	2.7	5.152	12	4.9	9.351	23.3	3	5.725	28.6	1.3	2.48	28.6
110	HC	51.4	2.9	5.642	3.7	7.198	8.3	6.1	11.868	21.4	2.6	5.058	5.2	2.1	4.085	19.4
111	HC	76	17.3	22.763	1.3	1.71	1.9	2.6	3.421	5.7	0.2	0.263	0.5	0.4	0.526	0.4
112	HC	54	6.3	11.666	3.7	6.851	6.9	7.4	13.704	14.5	1.3	2.407	4	0.7	1.296	6
113	HC	45.9	2.7	5.882	3.1	6.753	2.4	8.4	18.301	19	3.1	6.753	11.6	1.8	3.921	5.9
114	HC	54.8	3.9	7.116	2.2	4.014	5.1	3.6	6.569	9.4	1.8	3.284	6.7	1.1	2.007	4.9
115	HC	54.3	4.6	8.471	1.6	2.946	3.4	3.6	6.63	5.4	1.8	3.314	4.8	0.7	1.289	6.4
116	HC	59.5	3.9	6.51	7.6	12.773	27.6	12.5	21.008	40.2	2.2	3.697	9.9	13.4	22.521	37.6
117	HC	60	6.2	10.333	3.5	5.833	13	12.7	21.167	35.8	3.3	5.5	11.2	16.5	27.5	34.8
118	HC	44.2	6.2	11.6	4.3	9.316	12.1	6.1	11.324	17.4	3.6	6.812	10.96	4.2	7.234	13.2

C. Raw data of B cells and phenotype

No	Group	CD19	CD27 in CD19	CD38 in CD19	ICOSL in CD19	ICOSL in CD19CD27	ICOSL in CD19CD38	PDL1 in CD19	PDL1 in CD19CD27	PDL1 in CD19CD38	CD40 in CD19	CD40 in CD19CD27	CD40 in CD19CD38
1	CHB	20.7	10.628	84.878	5.361	7.92	8.11	2.272	8.72	5.92	97.551	92.2	89.9
2	CHB	4.9	17.241	74.074	5.882	8.12	6.97	13.333	7.5	7.34	97.959	91.3	92.4
3	CHB	6.2	50	70.967	6.451	7	8	9.677	11.5	9.2	91.304	94.1	95.5
4	CHB	9.1	25.274	67.032	2.197	3.7	2.8	3.296	8.3	3.1	73.62	50	56.5
5	CHB	7	50	68.571	8.571	14.9	10.6	5.714	7.5	9.3	31.428	41.4	43.2
6	CHB	4.1	19.512	56.097	9.756	37.5	13.6	14.634	50	13.3	65.853	50	57.1
7	CHB	4.9	16.326	63.265	2.04	5.4	3.4	0	0	0	96.078	90.6	96.2
8	CHB	5.3	22.641	86.792	1.886	7.4	3	5.66	13.8	4.3	85.106	55.6	83.2
9	CHB	11.1	17.117	78.378	2.702	1.2	3.2	1.801	3.8	1.9	98.373	98.3	98.6
10	CHB	10.9	30.275	76.146	5.504	1.2	7	0.917	2.3	1.2	96.226	90.7	95.6
11	CHB	11.3	37.168	57.522	3.539	7.9	6	8.849	11.2	8.4	98.901	96.7	98.4
12	CHB	11.8	19.491	83.898	4.237	7.5	4.3	3.389	7.3	3.8	94.202	86.7	94.9
13	CHB	4.5	42.222	62.222	8.888	11	12	11.111	16.7	15.2	89.655	90.7	92.9
14	CHB	16.2	35.802	44.444	1.234	2	2.7	1.851	3	4.1	97.972	96.2	96.2
15	CHB	8.2	52.439	57.317	7.317	6.8	12.2	10.975	16.9	16.5	98.901	98.3	98.4
16	CHB	4.3	41.86	60.465	9.302	13.2	15	6.976	17.5	15.1	86.111	85.5	86.3
17	CHB	6.2	27.419	82.258	4.838	11.7	4.9	1.639	4.3	2.5	91.666	83.6	91.1
18	CHB	5.2	19.23	82.692	3.846	8	4.7	3.846	6.9	2.1	95.744	80.4	95
19	CHB	7.1	38.028	63.38	1.408	2.7	2.1	7.142	2.2	1.9	98.648	95	97.8
20	CHB	6.1	21.311	75.409	6.557	9.2	7.3	8.196	7.6	7.8	96.703	95.3	97.6
21	CHB	2.3	45.454	72.631	4.347	4.2	8.12	4.347	3.4	7.1	96	82.5	93.2
22	CHB	3.3	23.272	69.322	6.06	8.7	7.33	3.03	0	5.4	92.857	81.9	96.6
23	CHB	8.4	42.857	58.333	7.142	4.9	9.9	3.571	3.8	3.6	98.837	97.8	98.4
24	CHB	7.3	24.657	75.342	13.698	6.7	17.4	6.849	5	7.1	98.837	99.2	99.4
25	CHB	11.6	24.137	80.172	0.862	1.3	1	1.869	4.7	2	99.145	97.3	99.3
26	CHB	10.9	21.1	82.568	3.669	2.9	5	1.904	2.8	1.7	99.107	96.8	98.8
27	CHB	11.9	38.655	64.705	2.542	5.1	3.6	5.05	6.2	5.4	98.058	91.2	97.5
28	CHB	5.8	48.275	67.241	4.545	5.2	7.8	6.451	10.3	9.3	92.857	100	97.4
29	CHB	12	23.33	78.33	2.439	4.4	3.1	1.869	6.2	2.7	82.758	79.8	84.5
30	CHB	7.4	22.972	79.729	2.564	3.6	2.6	1.369	3.8	1.5	100	100	100
31	CHB	12.4	29.032	80.645	4.761	1.7	5.3	2.325	3.5	2.4	97.6	93.4	97.6
32	CHB	20	29.5	71.5	6.5	1.4	7.3	0	0	0	82.467	79.5	84.8
33	CHB	7.7	27.273	77.922	5.194	9.5	6.9	4.464	8.2	5.5	93.67	86.6	94.7
34	CHB	3.7	29.729	64.864	7.894	9.5	10.9	4.44	8.7	6.9	96.296	100	98.5
35	CHB	3	33.333	0.7	16.129	25	23.3	8.108	5.4	11	100	96.6	98.5
36	CHB	10.4	35.576	38.461	3.738	7	8.6	8.849	13.8	17.5	98.924	96.9	98.3
37	CHB	6.1	49.18	62.295	12.121	18.8	18.3	15.789	30.8	23.2	83.582	77.4	82.3
38	CHB	7.5	24	68	12.82	24.1	14.2	3.658	6.8	4.4	97.368	95.3	97.4
39	CHB	3.1	38.709	61.29	6.06	5	6.1	5.882	7.7	7.5	87.5	91.3	97.1
40	CHB	12.6	29.365	70.634	0.793	1	1.1	2.38	1.2	2.5	97.58	94	97.3
41	CHB	12.6	29.365	70.634	0.793	1	1.1	2.38	1.2	2.5	97.58	94	97.3
42	CHB	8.9	26.966	80.898	1.123	0.8	1.7	3.37	0.5	3.2	87.671	100	92.5

43	CHB	11.4	32.456	82.456	3.478	4.9	3.8	0.934	0.7	0.7	91.111	96.6	97.9
44	AsC	10.5	29.123	70.123	3.912	4.03	5.6	4.245	7.5	5.27	94.239	84.2	90.2
45	AsC	14.3	20.2797	79.7202	11.888	5.5	14.6	15.1724	29.9	13.2	96.5277	83.7	95.2
46	AsC	5.4	44.444	56.6037	7.407	4.4	10.6	4.838	7.7	3.4	86	72	83.6
47	AsC	6.6	25.757	83.333	6.06	1.2	6.8	12.121	13.3	9.3	92.424	96.5	98
48	AsC	9.9	25.252	68.686	4.04	2.5	4.8	3.03	2.6	3.1	98.165	95.2	97.8
49	AsC	10.9	30.275	76.146	5.504	1.2	7	0.917	2.3	1.2	96.226	90.7	95.6
50	AsC	12.8	34.375	45.875	2.343	2.2	4.8	3.125	3.6	4.3	94.531	91.5	93.5
51	AsC	5.8	22.413	74.137	5.172	4.3	5.4	5.172	12.1	4.6	91.304	75.4	93.5
52	AsC	15.4	16.233	83.116	5.194	6.3	5.4	3.896	8.6	4.3	99.447	98.2	99.2
53	AsC	4.5	37.777	55.555	2.222	4.4	3.6	6.667	10.3	9.3	83.72	79.2	76.2
54	AsC	10.3	24.271	72.815	1.941	3.2	1.9	3.883	14.6	3.8	97.872	91.3	97.2
55	AsC	7.3	27.397	60.273	2.739	4.6	3.3	2.739	8.5	2.8	98.809	96.7	98.6
56	AsC	20	28	65	3.5	4.1	5	3	5.7	4.7	98.412	97.6	98.5
57	AsC	12	10.833	84.166	2.5	5	3.3	3.389	4.7	4.5	100	100	99.8
58	AsC	7.2	22.222	75	4.166	5	4.7	5	11.1	5.1	97.368	88.7	95.9
59	AsC	4.7	46.808	59.574	2.127	1.7	4.6	4.255	6.9	6.5	93.333	87.8	90.6
60	AsC	7.2	16.666	76.388	2.777	9.5	4	5.555	11.7	6.1	98.461	92	98
61	AsC	7.6	22.368	67.105	0	2.9	1	2.777	6.4	4.5	96.969	88.9	96.3
62	AsC	7.9	30.379	54.43	2.5	5.2	4.5	4.347	5.2	4.5	94.285	86.9	93.5
63	AsC	7	21.428	70	2.857	2.8	3.4	7.142	13.6	7.5	92.537	90.7	91.8
64	AsC	8.1	35.802	67.901	3.75	5.5	5.7	3.658	3.3	3.5	83.695	70.4	92
65	AsC	7	32.857	72.857	2.898	0	4.1	2.941	4.9	2.9	74.193	99.2	82
66	AsC	9.3	47.311	65.591	2.197	2.4	3.8	2.272	1	1.9	88.349	71.1	90.9
67	AsC	3.4	41.176	79.069	13.953	29.4	17.2	15.217	27.3	17.7	80.434	84.4	78.3
68	AsC	17.6	37.5	64.204	4.545	3	6.3	1.648	1.2	2.3	99.45	100	99.3
69	AsC	6.9	23.188	78.26	1.408	0	2.4	1.176	0	2.1	98.701	98.7	98.9
70	AsC	15.2	15.78	79.605	3.947	5.8	4.8	2.5	4.8	2.9	84.722	78.6	87.6
71	AsC	19.4	30.412	76.804	5.128	2.2	6.7	3.246	6.1	8.7	62.5	63.8	72.3
72	AsC	9.5	36.842	75.789	5.263	3.1	5.7	4.819	8.8	5.2	62.025	56.5	66.4
73	AsC	19.1	21.465	68.062	1.047	0	1.2	1.63	2.3	2.2	98.802	96.8	99.3
74	AsC	0.7	42.857	57.142	0	0	5.3	0	12.5	6.3	83.333	75	91.7
75	AsC	11.6	36.206	42.241	4.31	6.7	9	5.072	7.1	9	99.242	99.4	98.7
76	AsC	5.7	24.561	59.649	3.448	6.2	4.2	4.819	11.3	5.7	95.522	90.7	97.5
77	AsC	6.3	23.809	65.079	6.153	13	9.8	7.142	12.3	9.5	97.368	93.7	97.9
78	AsC	19.1	21.465	57.591	5.699	8.4	8.5	5.988	7.5	8.6	98.378	100	99.6
79	AsC	9	22.222	71.111	2.222	6.7	2.6	2.298	3.7	2.4	97.752	99	100
80	AsC	9.4	15.957	85.106	1.063	1.3	1	3.191	5.1	2.7	98.765	93.8	98.3
81	AsC	19	21.578	71.578	1.578	3.2	2.2	2.105	3.6	1.6	93.37	89.1	94.6
82	AsC	8.5	18.823	81.176	4.705	7.4	4.8	2.352	4.1	2.7	96.47	90	97
83	AsC	8.1	35.802	77.777	2.469	1.3	2.2	0	1.8	0.3	96.666	92.5	97.8
84	AsC	16.5	35.757	72.727	1.212	0.3	1.7	0	0	0	97.452	96.6	98.1
85	AsC	12.3	26.016	69.918	6.504	3.8	3.4	1.754	1.8	1.3	93.495	96.5	98.4
86	HC	12.4	20.967	79.032	29.838	10.2	33.7	1.612	4.5	1.5	97.58	84.4	96.7
87	HC	4.4	29.545	65.909	9.09	9	8.2	2.272	4.3	2	86.538	78.1	87.6
88	HC	4.6	17.391	69.565	2.173	2.7	3.3	0	1.7	0.5	92.592	87.5	92.6
89	HC	6.6	36.363	71.212	10.606	10.5	10.7	17.647	28	16.9	95.081	90.2	94.6
90	HC	5.4	31.481	66.667	5.556	3.4	5.4	1.851	4.9	2.9	93.442	89	92.5
91	HC	16.7	26.347	64.67	2.395	3.2	3	2.395	5.6	1.9	99.408	98.8	99
92	HC	6.1	22.95	65.573	8.064	13.3	12.9	10.769	9.4	14	98.666	97.8	98.4
93	HC	13.3	18.796	60.902	1.503	2.5	2.3	1.503	2.2	2.2	95.419	84.3	96
94	HC	10.5	46.666	56.19	2.857	3.9	5.3	3.809	2.1	5.2	92.035	91.9	92
95	HC	14.4	29.166	68.75	8.333	5.2	10.9	5.555	6.4	5.3	90.322	83.3	92
96	HC	15.1	15.231	76.158	12.582	2.6	13.6	2.04	2.1	2.2	99.358	98	99.2
97	HC	5.9	32.203	69.491	18.644	21.2	22.3	17.543	25	19.5	96.491	96.1	97.5
98	HC	5.2	51.923	69.23	8.064	5.9	10.2	5.172	4.2	5.8	69.863	55.9	85.6
99	HC	6.8	54.41	47.058	8.45	4	11.3	0	0	0	71.951	71.7	78.3

100	HC	11.6	31.034	77.586	20.689	17.7	26.8	5.319	19.4	6.6	83.928	93.4	86.8
101	HC	13.7	29.927	80.291	4.929	4.7	6.1	1.492	0.5	1.6	86.667	89.9	88.8
102	HC	6.2	43.548	61.29	5.714	0.8	8	1.47	0.8	1.1	86.956	90.7	93.3
103	HC	9.7	16.494	84.536	18.095	3.8	23.2	2.29	1.2	2.1	93.965	89.9	94.2
104	HC	6.9	39.13	79.71	4.285	3.1	5.3	3.26	6.6	3.2	98.507	95.1	98.8
105	HC	5.4	35.185	77.777	3.703	1.1	3.5	1.867	1.8	1.1	96.551	95.1	97.7
106	HC	4.7	31.914	68.085	4.166	8.3	5.8	4.081	7.7	3.9	96.153	100	99.4
107	HC	9	3.444	78.888	3.26	2.7	4.1	0.961	1.5	1.1	100	98.9	100
108	HC	3.9	58.974	41.025	0	1.8	2.6	0	2.4	1.9	87.234	90.7	92
109	HC	8.4	16.666	67.857	4.705	8.6	5.5	1.162	1.4	1.7	94.565	99.7	94.4
110	HC	5.8	48.275	65.517	1.724	1.4	1.5	1.724	1.3	1.7	96.875	92.9	94.3
111	HC	14	35	64.285	2.142	0.4	3	1.176	1	1.6	93.902	98.1	95.6
112	HC	8.5	30.588	75.294	1.176	0	1.6	1.176	2	1.4	99.056	98.6	99.2
113	HC	8.8	40.909	65.909	3.409	6.2	4.8	4.545	7.9	4.6	89.523	86.9	96.4
114	HC	9.1	29.67	76.923	6.593	9.5	7.2	4.494	10.2	5	94.791	86.7	94.2
115	HC	8.4	32.142	65.476	1.19	2.3	1.8	2.898	5.8	4.4	96.47	98.2	97.6
116	HC	4.6	50	36.956	34.782	26.2	51.5	36.956	31.4	59.1	100	99.4	100
117	HC	11.3	14.159	60.061	34.513	29.2	40.5	40	37	56.9	97.101	96.9	98.9
118	HC	16.6	29.518	77.108	9.638	15	30.7	8.849	19.2	10.1	99.019	97.2	99

D. Cytokines expression in the serum

No	Group	IL-4 (pg/ml)	IL-21 (pg/ml)	IL-6 (pg/ml)	IFN-r (pg/ml)
1	CHB	3	2834	12.1	49.6
2	CHB	1.9	1169.7	6.4	28
3	CHB	2.1	742.8	14.5	23.5
4	CHB	6.6	1518.9	5.1	251.8
5	CHB	2.2	5294.2	147.1	152.8
6	CHB	4	4490.3	62.8	35.2
7	CHB	7.2	1210	39.3	71.5
8	CHB	2	2745	6.5	39
9	CHB	3.4	2492.4	10.3	65.2
10	CHB	0.8	610.5	5.5	25.5
11	CHB	1.2	2205.7	8.8	29.2
12	CHB	2.1	5167.8	13.7	27
13	CHB	4.1	2179.7	5.8	182.6
14	CHB	1.7	832.1	3.9	47.7
15	CHB	6.3	799.6	1.8	20.7
44	AsC	5	1926.5	14.2	11.7
45	AsC	1.4	1167.8	16.2	344.1
46	AsC	1.1	4609.4	7.6	85
47	AsC	0.7	1021	23.9	29.3
48	AsC	3.6	1510.6	3.1	18.8
49	AsC	1	1231.3	16	7.9
50	AsC	0.7	1332.2	3.4	27.4
51	AsC	1.1	430	7.8	16.7
52	AsC	10	1887.6	1.9	6.7
53	AsC	3.1	1875.5	6.5	19
54	AsC	2.3	3297.3	4.8	14.2
55	AsC	2.6	450.5	2.3	17.5
56	AsC	3.1	1581.3	80.5	18.8
57	AsC	3.3	1907.3	9.4	36.5
58	AsC	2.2	659.6	12.9	45.4
86	HC	1.5	1606.1	16.4	32.6
87	HC	9.1	223.4	13.3	31.2
88	HC	3.9	1741.7	19.9	68.9
89	HC	14.6	1979.8	13.2	0

90	HC	8.8	0	7.9	10.4
91	HC	5.6	0	18.6	19.5
92	HC	0.2	72.8	20.1	61.6
93	HC	7.5	656.1	16.2	7.8
94	HC	4.2	1411.4	9.8	28.7
95	HC	7.4	802.3	17.9	29
96	HC	0.4	746.6	18.4	16.6
97	HC	1.9	1699.5	16	27.2
98	HC	1.3	1063.5	7.5	26.7
99	HC	0.8	1473.8	10.1	22.5
100	HC	0.4	1708.7	15.9	19.9
101	HC	0.1	707.5	9.8	0
102	HC	1.7	198.6	13.8	19.6
103	HC	1.2	978.6	8.2	0
104	HC	3.2	0	9.1	33.1
105	HC	6.1	0	11.5	0

E. Normalized expression of genes by ct value of Real-time PCR

BCL-6	normal CD4	HBV CD4	normal Tfh	HBV Tfh
lot 1#	1	2.384	2.038	8.22
lot 2#	1	4.462	3.653	23.541
lot 3#	1	2.741	4.203	13.407
lot 4#	1	3.587	3.194	11.213
lot 5#	1	3.005	2.657	7.319
lot 6#	1	5.503	3.632	13.573

CXCR5	normal CD4	HBV CD4	normal Tfh	HBV Tfh
lot 1#	1	1.982	7.278	33.217
lot 2#	1	3.421	16.209	27.123
lot 3#	1	2.855	9.178	15.482
lot 4#	1	1.965	7.249	13.261
lot 5#	1	3.33	2.474	6.895
lot 6#	1	2.722	16.926	41.306

IL-4	normal CD4	HBV CD4	normal Tfh	HBV Tfh
lot 1#	1	4.645	11.526	65.75
lot 2#	1	4.691	12.263	30.584
lot 3#	1	4.731	10.863	63.472
lot 4#	1	4.763	13.72	30.319
lot 5#	1	4.897	11.524	11.267
lot 6#	1	4.533	14.309	30.78

IL-21	normal CD4	HBV CD4	normal Tfh	HBV Tfh
lot 1#	1	5.831	24.063	27.921
lot 2#	1	1.869	1.761	8.08
lot 3#	1	3.364	10.336	30.782
lot 4#	1	4.131	2.111	35.056
lot 5#	1	5.584	2.505	6.887
lot 6#	1	2.223	14.123	29.251

IL-6R	normal CD4	HBV CD4	normal Tfh	HBV Tfh
lot 1#	1	1.162	3.002	5.15
lot 2#	1	3.953	3.099	1.94
lot 3#	1	3.559	2.412	3.911
lot 4#	1	1.646	1.709	3.769
lot 5#	1	2.809	0.877	2.786
lot 6#	1	2.623	5.507	11.3

F. Ig and chemokine secretion in the culture cells

IgM ng/ml	Health CXC5-CD4+ CD19+	HBV CXC5-CD4+ CD19+	Health CXC5+CD4+ CD19+	HBV CXC5+CD4+ CD19+
	76.36	468.0495	39.5768	909.77
	89.485	453.0497	76.5872	865.395
	83.921	458.423	235.1	905.21

IgG ng/ml	Health CXC5-CD4+ CD19+	HBV CXC5-CD4+ CD19+	Health CXC5+CD4+ CD19+	HBV CXC5+CD4+ CD19+
	654.262	2093.5	621.228	2474.87
	646.274	1598.54	606.946	2693.54
	543.428	1673.42	779.48	3201.22

XCL1 pg/ml	Health CXC5-CD4+ CD19+	HBV CXC5-CD4+ CD19+	Health CXC5+CD4+ CD19+	HBV CXC5+CD4+ CD19+
	15.2367	11.5015	46.921	75.653
	10.1959	18.3742	63.271	78.2775
	16.3316	4.86575	50.431	87.806

IL-4 (pg/ml)	Health CXC5+CD4+ CD19+	HBV CXC5+CD4+ CD19+
	2.6	3.2
	1.1	6.2
	1.9	6

IL-21 (pg/ml)	Health CXC5+CD4+ CD19+	HBV CXC5+CD4+ CD19+
	382.4	792
	105	632
	211.6	915

IL-6 (pg/ml)	Health CXC5+CD4+ CD19+	HBV CXC5+CD4+ CD19+
	1045.8	2374.1
	1178.1	1250.2
	1073.3	1031.51

G. Autoantibodies expression in patients

No	Group	ANA	AMA	ASMA	LKM	Nrnp/SM	Sm	ss-A	ss-B	SCL-70	Jo-1
		21	2	2	0	9	5	18	12	9	6
1	IA								+		
2	IA						+				
3	IA	+				+		+			
4	IA										
5	IA										
6	IA									+	
7	IA										
8	IA	+				+			+		
9	IA								+		
10	IA								+		

11	IA	+				+	+			
12	IA	+		+						
13	IA						+			
14	IA							+		
15	IA						+			
16	IA	+					+	+		
17	IA									
18	IA						+			
19	IA									
20	IA							+		
21	IA									
22	IA									
23	IA									
24	IA	+	+				+			
25	IA							+		
26	IA									
27	IA							+		
28	IA							+		
29	IA									
30	IA									
31	IA								+	
32	IA									
33	IA						+			
34	IA						+			
35	IA	+			+					+
36	IA									
37	IA									
38	IA	+			+				+	
39	IA						+			
40	IA									
41	IA									
42	IA									
43	IT	+			+					
44	IT	+	+				+			
45	IT	+			+					
46	IT	+				+	+			
47	IT									
48	IA							+		
49	IT									
50	IT				+	+				
51	IT	+					+			
52	IT								+	
53	IT									
54	IT							+		
55	IT									+
56	IT					+				
57	IT									
58	IT						+			
59	IT									
60	IT	+		+						+
61	IT									
62	IT	+					+			
63	IT	+					+		+	
64	IT									
65	IT									
66	IT									+
67	IT				+					
68	IT									
69	IT									

70	IT					
71	IT					
72	IT					
73	IT		+			
74	IT			+		
75	IT	+				+
76	IT				+	
77	IT				+	
78	IA	+				
79	IT	+			+	
80	IT					
81	IT	+		+		
82	IT	+			+	
83	IT					
84	IT					+
85	IT					
