# Supplementary material for: Production of Autoantibodies in Chronic Hepatitis B Virus Infection Is Associated with the Augmented Function of Blood CXCR5+CD4+ T Cells
Source: PLoS One. 2016 Sep 9;11(9):e0162241. doi: 10.1371/journal.pone.0162241 (PMC5017876; doi:10.1371/journal.pone.0162241)

S1 Fig. Fluorescence activating cell sorter (FACS) analysis of the frequency and phenotype of peripheral blood CXCR5^{+}CD4^{+} T cells

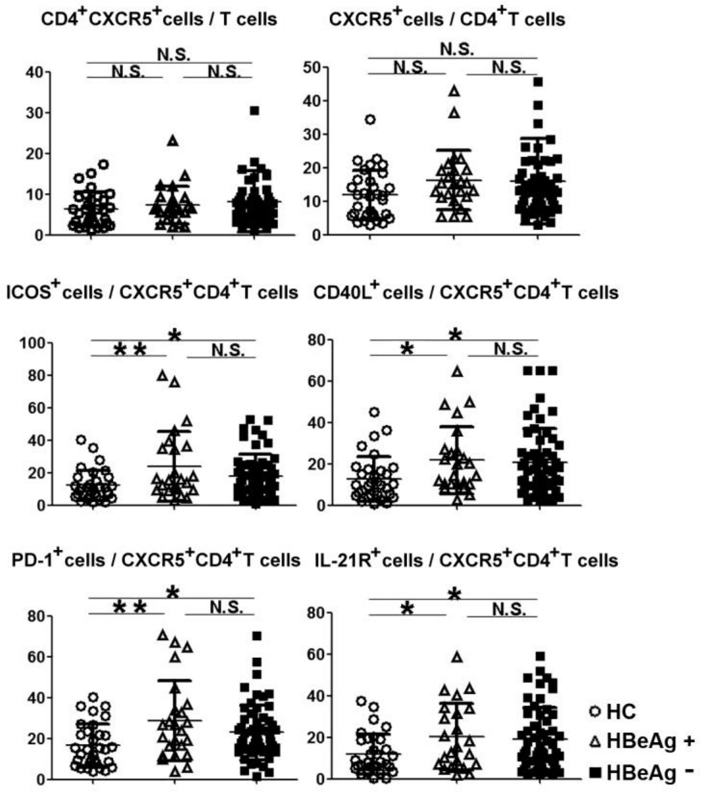

Supplement: S1 Fig — The frequencies of total blood CXCR5+CD4+T cells and ICOS-1+/ PD-1+/ CD40L+/IL-21R+CXCR5+CD4+T cells were compared among health control (HC, n = 33), HBeAg positive patients with chronic HBV infection (HBeAg +, n = 29), and HBeAg negative patients with chronic HBV infection (HBeAg -, n = 56). Means and standard deviations of cell frequency are shown. *, P < 0.05, **, P < 0.01, NS, not significant (P > 0.05) (Student's t-test). (PDF) [file pone.0162241.s002.pdf]
