# Supplementary material for: Production of Autoantibodies in Chronic Hepatitis B Virus Infection Is Associated with the Augmented Function of Blood CXCR5+CD4+ T Cells
Source: PLoS One. 2016 Sep 9;11(9):e0162241. doi: 10.1371/journal.pone.0162241 (PMC5017876; doi:10.1371/journal.pone.0162241)
Supplement: S1 Table — (PDF) [file pone.0162241.s003.pdf]

Supplementary table 1. Pearson correlation coefficients for frequencies of blood cell types in patients with chronic HBV infection

Cell type（%）	$CXCR5^+CD4^+$ in T	$ICOS^+$ in $CXCR5^+CD4^+$ T	$CD40L^+$ in $CXCR5^+CD4^+$ T	$PD1^+$ in $CXCR5^+CD4^+$ T
B cell in PBMC	-0.112	-0.159	-0.105	-0.135
$ICOSL^+$ in B	-0.057	0.285**	0.260*	0.201
$CD40^+$ in B	-0.153	0.172	0.277*	0.214
$PDL1^+$ in B	0.285**	0.308**	0.332**	0.284**

NOTE. Coefficients range between -1 and 1. Negative correlations are indicated by values from -1 to 0, and positive correlations are indicated by values from 0 to 1. *, P ＜0.05, 2-tailed, **, P ＜ 0.01, 2-tailed.
