# Supplementary material for: Production of Autoantibodies in Chronic Hepatitis B Virus Infection Is Associated with the Augmented Function of Blood CXCR5+CD4+ T Cells
Source: PLoS One. 2016 Sep 9;11(9):e0162241. doi: 10.1371/journal.pone.0162241 (PMC5017876; doi:10.1371/journal.pone.0162241)
Supplement: S2 Table — (PDF) [file pone.0162241.s004.pdf]

Table 3. Pearson correlation coefficients for frequencies of blood cells and clinical data in patients with chronic HBV infection

Cell type（%）	ALT	AST	HBV DNA
$CXCR5^+CD4^+$ in T cell	0.413**	0.463**	0.229*
$CXCR5^+$ in $CD4^+$ T cell	0.425**	0.469**	0.249*
$CD19^+$ B cell in PBMC	-0.135	-0.117	-0.200
$CD27^+$ B cell	0.047	0.040	-0.015
$CD27^+CD38^+$ B cell	0.315**	0.367**	0.212
$CD40^+$ B cell	-0.426**	-0.464**	-0.154
$CD40^+$ in $CD27^+$ B cell	-0.494**	-0.545**	-0.213
$CD40^+$ in $CD27^+CD38^+$ B cell	-0.423**	-0.458**	-0.091

NOTE. Coefficients range between -1 and 1. Negative correlations are indicated by values from -1 to 0, and positive correlations are indicated by values from 0 to 1. *, $P < 0.05$, 2-tailed. **, $P < 0.01$, 2-tailed.
